# Bond strength of lithium disilicate after cleaning methods of the remaining hydrofluoric acid

**DOI:** 10.4317/jced.56412

**Published:** 2020-02-01

**Authors:** Daniela-Micheline dos Santos, Sandro-Basso Bitencourt, Emily-Vivianne-Freitas da Silva, Adaias-Oliveira Matos, Georgia-de Castro Benez, Elidiane-Cipriano Rangel, Aldiéris-Alves Pesqueira, Valentim-Adelino-Ricardo Barão, Marcelo-Coelho Goiato

**Affiliations:** 1 DDS, PhD, Professor, Department of Dental Materials and Prosthodontics, Sao Paulo State University (UNESP), Araçatuba, Sao Paulo, Brazil; 2 DDS, MSc, PhD student, Department of Dental Materials and Prosthodontics, Sao Paulo State University (UNESP), Araçatuba, Sao Paulo, Brazil; 3 DDS, MSc, PhD Student, Department of Prosthodontics and Periodontology, University of Campinas (UNICAMP), Piracicaba, Sao Paulo, Brazil; 4 DDS, Department of Dental Materials and Prosthodontics, Sao Paulo State University (UNESP), Araçatuba, Sao Paulo, Brazil; 5 PhD, Professor, Laboratory of Technological Plasmas, São Paulo State University (UNESP), Sorocaba, Sao Paulo, Brazil; 6 DDS, PhD, Professor, Department of Prosthodontics and Periodontology, University of Campinas (UNICAMP), Piracicaba, Sao Paulo, Brazil

## Abstract

**Background:**

Different ceramic surface cleaning methods have been suggested after the acid conditioning. The aim was to evaluate the effect of different protocols used to remove the remaining hydrofluoric acid on the shear bond strength (SBS) between lithium disilicate and resin cement.

**Material and Methods:**

Forty-four specimens of lithium disilicate (IPS e.max Press) were divided in 4 groups (n=11): group C (control, no treatment); group HF+S (5% hydrofluoric acid + silane); group HF+US+S (5% hydrofluoric acid + ultrasound cleaning + silane); group HF+PH+S (5% hydrofluoric acid + 37% phosphoric acid + silane). Scanning electron microscopy (SEM) and energy dispersive spectroscopy (EDS) were performed to characterize the surface morphology. The SBS test was performed on the resin/ceramic interface, and the failure mode was characterized. SBS values were submitted to 1-way ANOVA and the Tukey test (α=.05). The relation between surface treatment and failure modes was analyzed using the chi-squared test (α=.05).

**Results:**

The surface treatment type interfered in the shear strength (*p*<.001) and higher SBS values were observed for the groups HF+US+S (17.87 MPa) and HF+PH+S (16.37 MPa). The surface treatment did not influence the failure mode (*p*=.713). No fluorsilicate salts were observed after ultrasound cleaning.

**Conclusions:**

The utilization of ultrasound cleaning was an effective procedure to remove remaining fluorsilicate salts, promoting the highest SBS values.

** Key words:**Bond strength, ceramics, fluorsilicate, lithium disilicate, resin cement.

## Introduction

The clinical procedure of adhesive cementation of all-ceramic fixed prostheses involves the treatment of the dental substrate and the internal surface of the ceramic restoration to be installed (1,2). It is agreed that the use of hydrofluoric acid followed by the application of silane is the most effective surface treatment method for glass ceramics (2,3).

However, it is known that the conditioning of the surface with hydrofluoric acid produces insoluble fluorosilicate salts, which could decrease the molecular contact between the resinous cement and the ceramic, negatively affecting the adhesion process (4). Thus, different ceramic surface cleaning methods have been suggested after the acid conditioning, such as the use of an ultrasonic bath, 37.5% phosphoric acid, or even neutralization of the acid with substances such as sodium bicarbonate (5-9).

There is a great preoccupation with the use of hydrofluoric acid in the surface treatment of ceramics since, due to its toxicity and high corrosive power, it could be absorbed easily by the skin, blood, and bones, causing harmful effects to patients’ health (10-12). Also, the hydrofluoric acid could decrease the glass-ceramics flexural strength, increasing their susceptibility to fractures (4,13,14).

Therefore, this study aimed to characterize the morphology of different protocols used to remove the remaining hydrofluoric acid and to evaluate their effect on the shear bond strength (SBS) between lithium disilicate ceramic and resin cement. The null hypothesis tested was that the different protocols would not influence the SBS values.

## Material and Methods

Forty-four lithium disilicate glass-ceramic specimens (IPS e.max Press, Ivoclar Vivadent AG) were fabricated using the lost-wax technique from autopolymerizing acrylic resin discs (10 mm in diameter and 3 mm thick) (Duralay, Polidental)15. The surface of the lithium disilicate specimens were polished using sequential metallographic sandpapers (#320, #400, #600) (Carbimet 2, Buehler) in an automatic polishing machine (Aropol 2V, Arotec) at a velocity of 300 rpm. Subsequently, they were submitted to an ultrasound bath (UltraMet 2002; Buheler) in distilled water for 1 minute, followed by a bath in a 99.3% ethyl alcohol solution for 5 minutes, and another bath in distilled water for 1 minute (15).

The specimens were divided in four groups (n=11) according to the surface treatment employed: group C (control, no treatment); group HF+S (5% hydrofluoric acid + silane); group HF+US+S (5% hydrofluoric acid + ultrasound cleaning + silane); group HF+PH+S (5% hydrofluoric acid + 37% phosphoric acid + silane). Treatment protocols were performed according to the following: group C: no surface treatment was performed after the cleaning of the specimens; group HF+S: the surface was treated according to the resin cement manufacturer’s recommendation, being conditioned with 5% hydrofluoric acid (IPS Ceramic Etching Gel, Ivoclar Vivadent AG) for 20 seconds, subsequently washed with a spray jet and water for 30 seconds, dried with jets free of oil and humid + silanization with a thin layer of silane (Monobond S, Ivoclar Vivadent AG); group HF+US+S: conditioned with hydrofluoric acid as described in group 2 + an ultrasound bath in distilled water for 5 minutes + silanization as described in group 28; group HF+PH+S: conditioned with hydrofluoric acid as described in group 2 + conditioned with 37% phosphoric acid gel (Total Etch, Ivoclar Vivadent AG) for 60 seconds, subsequently washed with a spray jet and water for 30 seconds, dried with jets free of oil and humid + silanization as described in group 28.

One specimen from each group was used for the SEM (JSM 610LA; JEOL) analysis15. Micrographs were registered with an increase of 10,000×, in different stages: T0 (specimen without treatment), T1 (after treatment with hydrofluoric acid), T2 (after treatment with hydrofluoric acid followed by cleaning with ultrasound, T3 (after treatment with hydrofluoric acid followed by cleaning with 37% phosphoric acid) and T4 (after application of silane). The characterization of elementary chemical composition in small volumes was performed on the order of 1 µm³ through the energy dispersive spectroscopy (EDS) (15).

For the SBS test, cylinders of 5 mm of diameter × 2.5 mm of height were fabricated from universal composite resin (Z100; 3M-ESPE) from a laboratory silicone mold (ZetaLabor; Zhermack) with a perforation in the center. The composite resin was manipulated and inserted in the interior of the perforation with 2 mm-increments, and light-cured (Rembrandt Allegro LED Curing Light; Den-Mat) for 40 seconds. Each cylinder was cemented over the ceramic specimen surface using the light-cured resinous cement (Variolink Veneer, Ivoclar Vivadent AG) and a device (1000 g) was used to standardize the pressure during the cementation. After the careful removal of the excess cement with disposable applicators (KG Brush, KG Sorensen), a light-curing was performed for 60 seconds at four different points of the resin/ceramic interface, maintaining the weight in position (16).

Then, the SBS test was performed using a universal testing machine (Instron Model 4400 Universal Testing System, Instron Corporation), with a transversal velocity of 1 mm/min on the surface at the resin/ceramic interface. The load value until failure was determined in MPa. After the SBS test, the failure mode was evaluated through a stereomicroscope (V20 SteREO Descoberta, Carl Zeiss) with an increase of 150×, and divided in adhesive, cohesive, and mixed (17).

Data obtained from SEM and EDS analyses were compared visually. SBS values were submitted to one-way analysis of variance (ANOVA) and the Tukey test (α=.05) (SPSS version 20.0 – Statistical Package for the Social Sciences, IBM Corp). The relation between surface treatments and failure modes was analyzed utilizing the Chi-squared test (α=.05).

## Results

The type of surface treatment interfered in the shear strength (*p*<.001) ( Table 1). Higher SBS values were observed for groups HF+US+S (17.87 MPa) and HF+PH+S (16.37 MPa), which were statistically different from the other groups analyzed. The smallest SBS value was found for group C (3.84) ( Table 2).

**Table 1:**
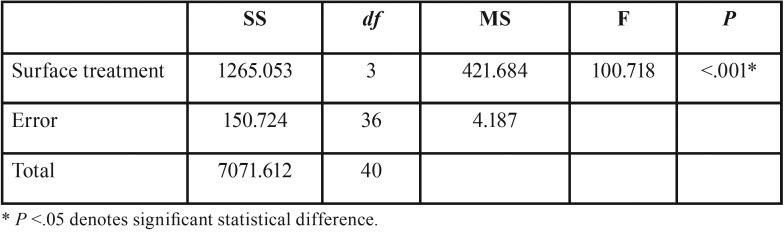
One-way ANOVA of SBS between lithium disilicate and resin cement for different surface treatments.

**Table 2:**
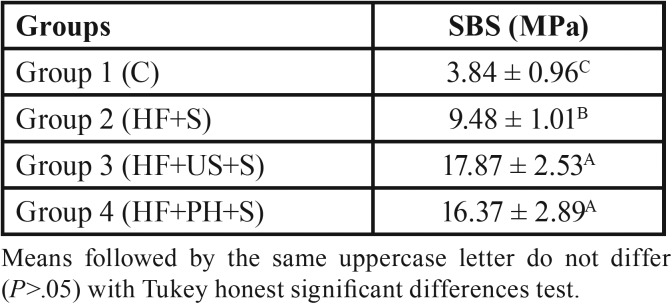
Mean values and standard deviation of SBS between ceramic and resin cement (MPa).

**Figure 1:**
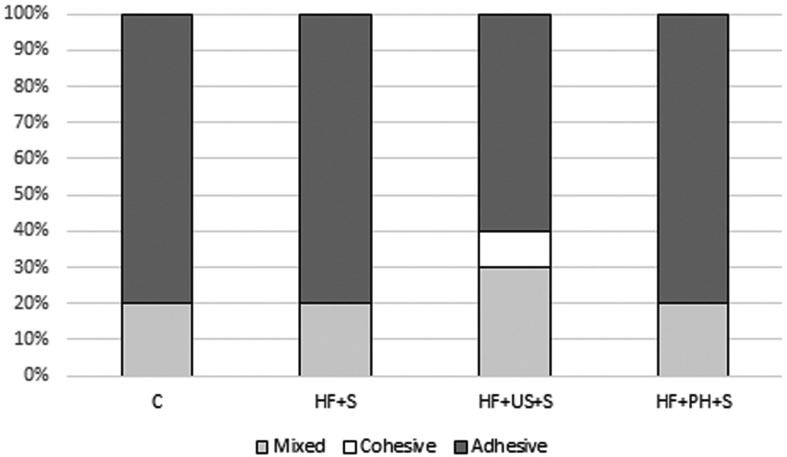
Percentage distribution of bond failure types (adhesive, cohesive, or mixed) between ceramic and resin cement in groups analyzed. Group C: no surface treatment; Group HF+S: 5% hydrofluoric acid + silanization; Group HF+US+S: 5% hydrofluoric acid + ultrasound + silanization; Group HF+PH+S: 5% hydrofluoric acid + 37% phosphoric acid gel + silanization.

Figure 1 illustrates the percentage distribution of bond failures between the ceramic and the resin cement for different surface treatments analyzed. The type of surface treatment did not influence the failure mode (Chi-squared test, *p*=.713).

**Figure 2:**
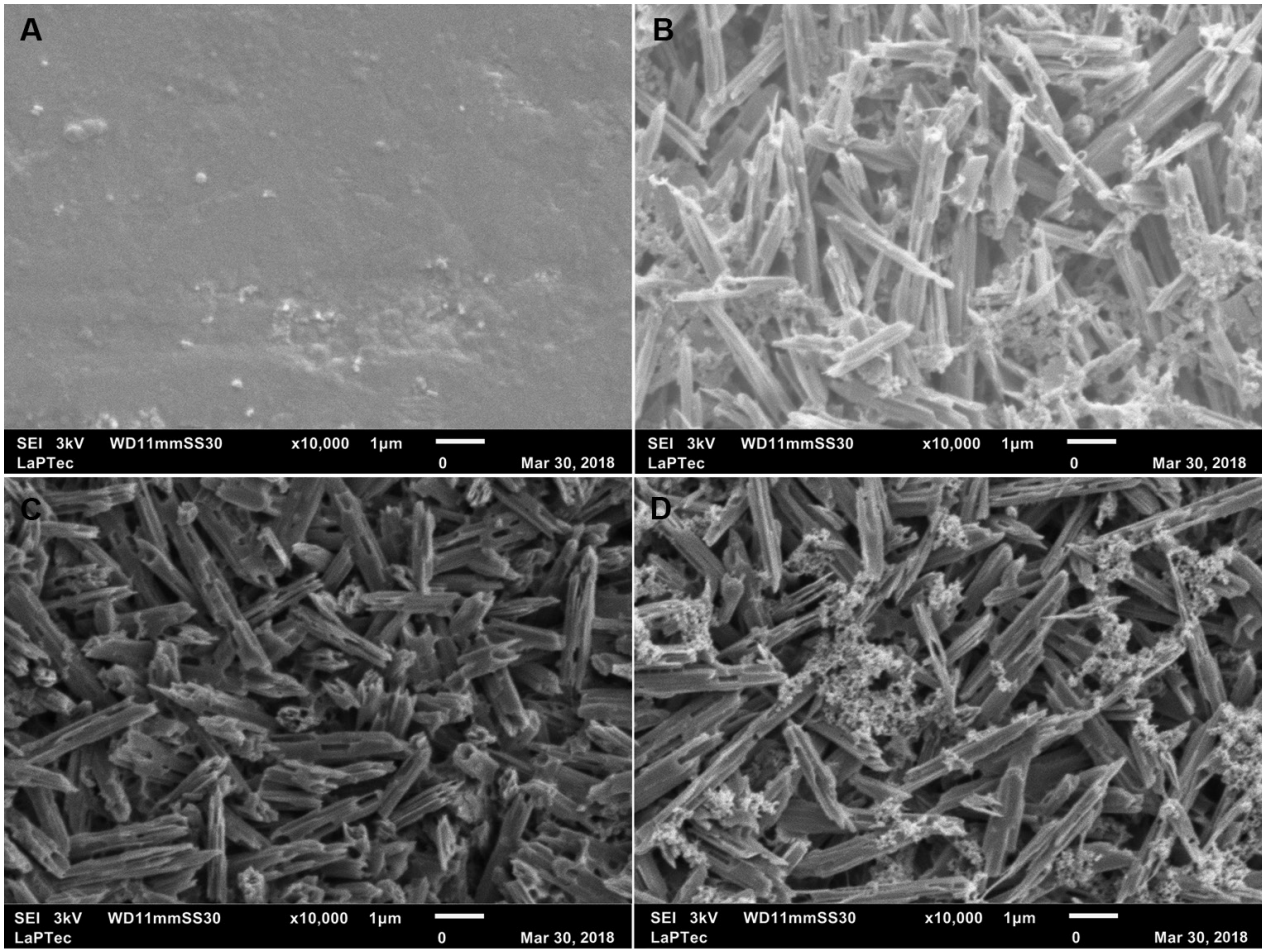
Representative SEM micrographs (10,000×) of ceramic specimens in periods: (A) T0 (specimen without treatment); (B) T1 (after treatment with hydrofluoric acid); (C) T2 (after treatment with hydrofluoric acid followed by cleaning with ultrasound); and (D) T3 (after treatment with hydrofluoric acid followed by cleaning with 37% phosphoric acid).

According to Figure 2 (T1 and T3), the dissolution of the glassy phase was observed with the exposition of lithium disilicate crystals, surrounded by fluorosilicate precipitates. In the period T2, the presence of exposed lithium disilicate crystals was observed.

**Figure 3:**
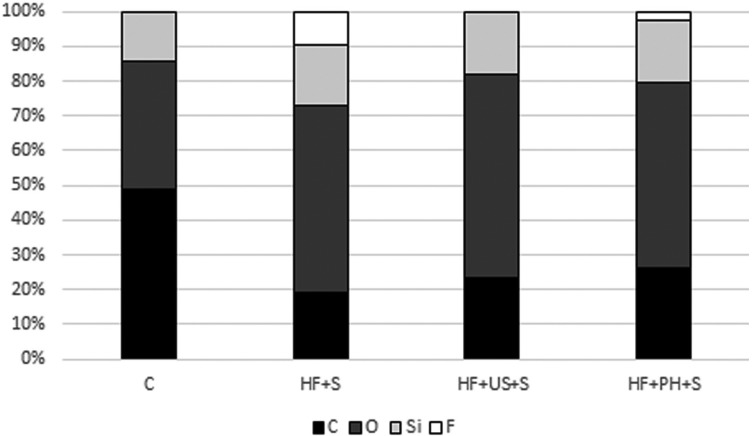
Percentage distribution of elements identified in the EDS of ceramic specimens analyzed in different stages. Group C: no surface treatment; Group HF+S: 5% hydrofluoric acid + silanization; Group HF+US+S: 5% hydrofluoric acid + ultrasound + silanization; Group HF+PH+S: 5% hydrofluoric acid + 37% phosphoric acid gel + silanization.

Through the EDS spectrum, high percentages of carbon (C) and oxygen (O), and smaller percentages of silicon (Si) can be observed for all the periods evaluated (Fig. 3). Fluorine (F) was verified only in the T1 and T3 periods.

## Discussion

Based on the results encountered, the null hypothesis was rejected since there was a statistical difference between the groups evaluated after the SBS test. In the micrographs obtained, it was also possible to verify that there were alterations on the surface of the specimens according to the treatment performed.

Since an adequate bond between the resinous cement and the ceramic restoration is originated from a satisfactory surface treatment (18), this study reached out to verify the influence of different surface treatments on the SBS between lithium disilicate and resin cement, by testing two different protocols of removal of the remaining hydrofluoric acid. It was observed that cleaning groups (groups 3 and 4) showed higher SBS results when compared to the control and the protocol (group 2) often used. The ultrasound cleaning after hydrofluoric acid application resulted in a surface without fluorosilicate precipitates and a higher SBS value.

Hydrofluoric acid selectively etches the glass-ceramic, promoting physical alteration of the surface and, consequently, micromechanical retention (1,2,6). Then, this ceramic is rinsed to remove the acid and a silane coupling agent is applied (4,8). However, acid residual could remain in the ceramic surface, affecting the adhesion process (2), flexural strength (8,9,19), and could also be toxic to the patient (10,12). Therefore, it is important to completely remove this acid from the surface. Besides, when composites are exposed to HF acid gel, water may penetrate spaces, filling them. As a result, a disorganization of the siloxane structure, formed from the condensation of intermolecular silanol groups, can occur (20).

The use of phosphoric acid did not completely remove the precipitates of fluorine deposited on the specimens’ surface, which was verified by the SEM images (Fig. 2D) and by the EDS test, which detected the presence of F on the surface (Fig. 3). Despite no significant statistical difference was verified, the presence of this sub-product, even in a small percentage could have influenced the SBS of the group HF+PH+S, which was smaller than the group HF+US+S ( Table 2).

The limitations of this study are that this is an *in vitro* study, needing more clinical studies to extrapolate these findings, also the SBS test used to evaluate the bond strength between cement and ceramic has been severely criticized for quite some time, since its results may not be consistent due to a non-uniform force distribution on the bond interface, causing cohesive failures in the substrate (21). However, we believe that it did not occur in the present study since the adhesive failure mode was the most prevalent in all the groups. In this way, the results of this study are important to show the presence of the hydrofluoric acid in the lithium disilicate surface, even though rinsed with water after the conditioning procedure. The cleaning procedure demonstrated to improve the bond strength, regardless of the type of cleaning.

## Conclusions

Based on the results encountered, the utilization of a cleaning procedure after the conditioning with hydrofluoric acid is extremely important. These procedures increased the bond strength between lithium disilicate and resin cement, especially after ultrasound cleaning.
